# Cytoprotective Effects of Agomelatine on Hepatic Ischemia–Reperfusion Injury in a Rat Model

**DOI:** 10.3390/ijms27073246

**Published:** 2026-04-02

**Authors:** Yilmaz Bilgic, Sami Akbulut, Oguzhan Yildirim, Onural Ozhan, Azibe Yildiz, Zeynep Erdemli, Mehmet Erman Erdemli, Adem Kose, Nigar Vardi, Yusuf Turkoz, Hakan Parlakpinar

**Affiliations:** 1 Department of Gastroenterology, Faculty of Medicine, Inonu University, 44280 Malatya, Türkiye; 2 Department of Surgery, Faculty of Medicine, Inonu University, 44280 Malatya, Türkiye; 3 Department of Pharmacology, Faculty of Medicine, Inonu University, 44280 Malatya, Türkiye; 4 Department of Histology, Faculty of Medicine, Inonu University, 44280 Malatya, Türkiye; 5 Department of Biochemistry, Faculty of Medicine, Inonu University, 44280 Malatya, Türkiye; 6 Department of Infectious Diseases and Clinical Microbiology, Faculty of Medicine, Inonu University, 44280 Malatya, Türkiye

**Keywords:** liver, ischemia–reperfusion injury, oxidative stress, agomelatine, antioxidant capacity, inflammation, apoptosis

## Abstract

Hepatic ischemia–reperfusion injury (IRI) is a major cause of liver damage and is characterized by oxidative stress, inflammatory signaling, and hepatocellular apoptosis. Aim: This study investigated the hepatoprotective effects of agomelatine (AGO) administered before ischemia or at the onset of reperfusion in a hepatic IRI model. Rats were allocated into four experimental groups: Sham, IRI, IRI+AGO, and AGO+IRI. Hepatic ischemia was induced by clamping the hepatic pedicle for 1 h followed by 1 h of reperfusion. AGO (20 mg/kg) was administered orally either before ischemia or at the onset of reperfusion. Oxidative stress markers, antioxidant enzymes, nitric-oxide-related parameters, cytokines, liver injury enzymes, and histopathological changes were evaluated. IRI increased oxidant markers and reduced antioxidant defenses. AGO treatment improved redox balance and antioxidant parameters in both treatment groups, with stronger antioxidant responses observed in the AGO+IRI group. Nitric oxide (NO)-related markers differed among groups, including changes in L-arginine, asymmetric dimethylarginine (ADMA), and symmetric dimethylarginine (SDMA) levels, and interleukin-6 (IL-6) levels decreased following AGO administration, particularly in the AGO+IRI group. Histopathological injury and caspase-3 expression were also attenuated in AGO-treated animals. AGO attenuates hepatic IRI by improving redox balance, modulating NO metabolism, and reducing IL-6–associated signaling and apoptosis, with stronger protection when administered before ischemia.

## 1. Introduction

Ischemic organ damage remains a major global health problem, contributing to significant morbidity and mortality in surgical, transplant, and critical care settings [1,2]. Among these conditions, hepatic ischemia–reperfusion injury (IRI) is a major challenge during liver transplantation (LT), hepatic resection, trauma surgery, and circulatory shock [3]. Interruption and restoration of hepatic blood flow trigger metabolic and immunologic events that compromise hepatocellular integrity. Clinically, hepatic IRI is closely associated with primary non-function (PNF) and early allograft dysfunction (EAD) after LT, both linked to increased complications and reduced graft survival [4,5,6]. Contemporary data indicate that PNF occurs at lower, center-dependent rates (approximately 1–8%), yet remains a severe complication often requiring urgent retransplantation. EAD is more frequent, with reported incidences of 20–44%, and represents a major determinant of short-term graft performance [7,8,9,10,11,12]. These observations highlight the early reperfusion period as a critical therapeutic window for interventions aimed at improving graft function.

Hepatic IRI is a multifactorial process with phase-dependent biochemical and molecular alterations [2,13,14,15,16,17,18]. Early reperfusion is characterized by mitochondrial dysfunction, oxidative stress imbalance, and activation of apoptotic pathways, whereas inflammatory infiltration and necrosis predominate later [18,19,20]. Excessive generation of reactive oxygen species (ROS) overwhelms endogenous antioxidant systems and disrupts cellular homeostasis [21,22]. In parallel, impaired nitric oxide (NO) bioavailability contributes to microcirculatory dysfunction and amplifies inflammatory and apoptotic signaling during reperfusion [23,24,25,26,27,28]. Accordingly, multiple strategies have been explored to attenuate oxidative and inflammatory injury, and antioxidant-based interventions have consistently reduced biochemical and histological damage in experimental hepatic IRI models [23,24,25,26,27,28].

Several antioxidant and cytoprotective agents—including N-acetylcysteine, melatonin, curcumin, resveratrol, S-adenosylmethionine, agomelatine (AGO), and NO–modulating compounds—have been investigated in hepatic IRI models [29,30,31,32,33]. AGO is a melatonin MT1/MT2 receptor agonist and a serotonin 2C receptor (5-HT 2C) antagonist, combining neurohormonal and antioxidant properties [34,35,36,37,38]. Nuclear melatonin receptors have been identified in hepatocytes, and melatonin exerts antioxidant and anti-inflammatory effects, particularly through MT1 receptor activation [39]. Accumulating evidence indicates that AGO can mimic several cytoprotective actions of melatonin and exhibit antioxidant and anti-inflammatory properties in diverse experimental models [40]. In this context, AGO is of interest as a repurposed compound with a pharmacologically distinct profile. It may represent a candidate intervention capable of modulating early IRI-related oxidative and inflammatory cascades within defined timing windows (pre-ischemia vs. onset of reperfusion) [1,41,42,43,44,45].

Despite these potential benefits, AGO has also been associated with rare but potentially severe hepatotoxicity in clinical use, including marked elevations in transaminases and, in exceptional cases, serious liver injury [46,47]. This dual hepatic safety profile underscores the need for a cautious, mechanistic evaluation of AGO in experimental settings.

Limited but growing experimental evidence supports the hepatoprotective potential of AGO in oxidative liver injury models. In antineoplastic- and paracetamol-induced hepatotoxicity models, AGO reduced thiobarbituric acid reactive substances (TBARS) accumulation and restored key antioxidant defenses, including reduced glutathione (GSH), superoxide dismutase, catalase (CAT), and glutathione peroxidase (GSHPx), thereby ameliorating histopathological damage [40,48,49,50]. However, the effects of AGO on hepatic IRI remain insufficiently characterized, and no comprehensive study has evaluated its protective efficacy in a rat hepatic IRI model. Moreover, reports of AGO-associated hepatotoxicity further emphasize the importance of balanced assessment [51,52].

Accordingly, the present study aimed to investigate the antioxidant, anti-inflammatory, and hepatoprotective effects of AGO administered either pre-ischemic (prophylactic) or at the onset of reperfusion (therapeutic) in an experimental hepatic IRI model. By integrating biochemical, histopathological, and immunohistochemical analyses, this study evaluates the timing-dependent protective potential of AGO and addresses a specific gap in the literature regarding its role in hepatic IRI.

## 2. Results

### 2.1. AGO Attenuates Oxidative Stress and Restores Antioxidant Defense in Hepatic IRI

To evaluate the effects of AGO on hepatic IRI, a rat hepatic IRI model was established by clamping the hepatic pedicle followed by reperfusion, and AGO was administered either before ischemia or at the onset of reperfusion. The experimental design and treatment protocols are described in detail in Section 4.2 (Materials and Methods).

#### 2.1.1. AGO Reduces Lipid Peroxidation and Improves Antioxidant Enzyme Activity

TBARS levels differed significantly among the four groups (H = 20.8, *p* < 0.001). Dunn’s post-hoc analysis demonstrated that the IRI group differed significantly from both the IRI+AGO group (*p* = 0.0167) and the AGO+IRI group (*p* < 0.001), whereas the remaining pairwise comparisons were not statistically significant (adjusted *p* > 0.05). These findings support that the increase in lipid peroxidation induced by IRI was reduced with AGO administration.

GSH levels showed a significant difference among groups (H = 22.8, *p* < 0.001). In post-hoc analysis, the IRI group differed significantly from the AGO+IRI group (*p* < 0.001), while the other comparisons were not statistically significant (e.g., Sham vs. IRI *p* = 0.120; IRI vs. IRI+AGO *p* = 0.090; Sham vs. AGO+IRI *p* = 0.090). These findings suggest that antioxidant capacity improved particularly with pre-ischemic AGO administration (AGO+IRI).

CAT activity differed significantly among groups (H = 22.8, *p* < 0.001). Post-hoc analysis indicated that the IRI group differed significantly from the AGO+IRI group (*p* < 0.001), whereas other pairwise comparisons were not statistically significant (adjusted *p* > 0.05). These results indicate that CAT activity was most prominently restored in the AGO+IRI protocol.

GSHPx levels showed a significant difference among groups (H = 21.4, *p* < 0.001). Post-hoc analysis revealed that the IRI group differed significantly from both the IRI+AGO group (*p* = 0.008) and the AGO+IRI group (*p* < 0.001), while the remaining comparisons were not statistically significant (adjusted *p* > 0.05). These findings indicate that AGO enhanced the antioxidant enzyme response, with a more pronounced effect observed in the AGO+IRI protocol.

Total nitrite levels differed significantly among groups (H = 23.2, *p* < 0.001). Post-hoc analysis showed that the IRI group differed significantly from the AGO+IRI group (*p* < 0.001). Borderline differences were observed for Sham vs. AGO+IRI (*p* = 0.05) and IRI vs. IRI+AGO (*p* = 0.05). These findings indicate a marked increase in nitrite levels particularly in the AGO+IRI protocol.

Total Antioxidant Status (TAS) levels showed a significant difference among groups (H = 15.1, *p* = 0.002). Post-hoc analysis demonstrated that the IRI group differed significantly from the AGO+IRI group (*p* = 0.001), whereas other pairwise comparisons were not statistically significant (adjusted *p* > 0.05; Sham vs. IRI *p* = 0.05, borderline). These results suggest that total antioxidant capacity improved most prominently with AGO+IRI.

Total oxidant status (TOS) levels differed significantly among groups (H = 21.2, *p* < 0.001). Post-hoc analysis indicated that the IRI group differed significantly from the AGO+IRI group (*p* < 0.001), while other comparisons were not statistically significant (adjusted *p* > 0.05). These findings support that oxidant load was markedly reduced particularly in the AGO+IRI protocol.

**Figure 1:**
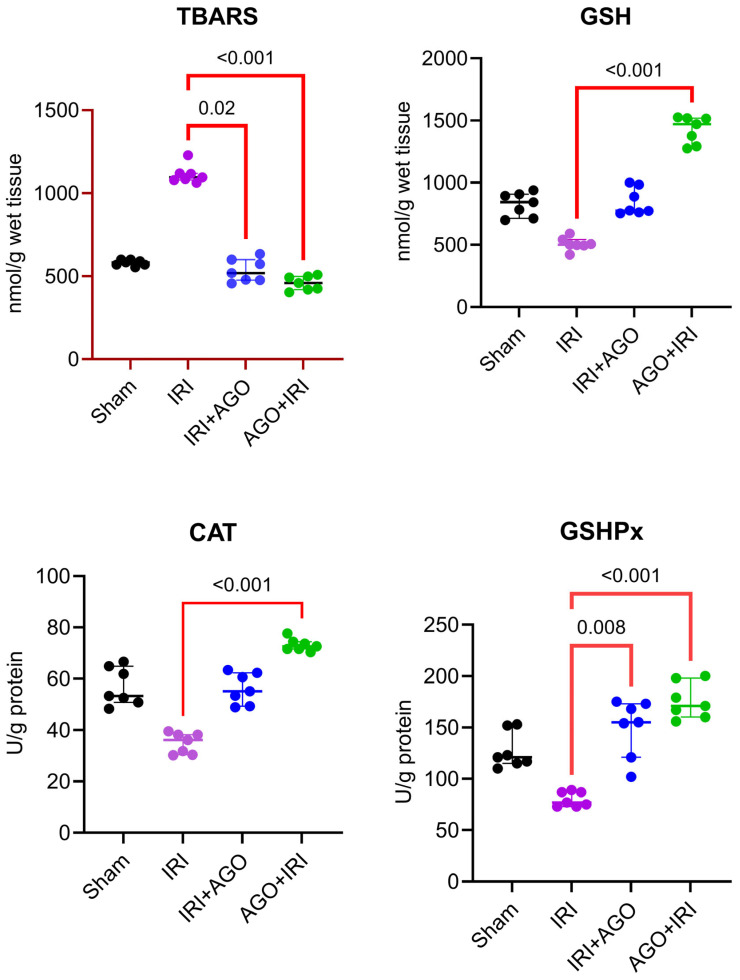
Individual data points of tissue oxidative and antioxidant markers across experimental groups (TBARS, GSH, CAT, and GSHPx). Overall group differences were assessed by Kruskal–Wallis test, followed by Dunn’s multiple-comparisons test.

**Figure 2:**
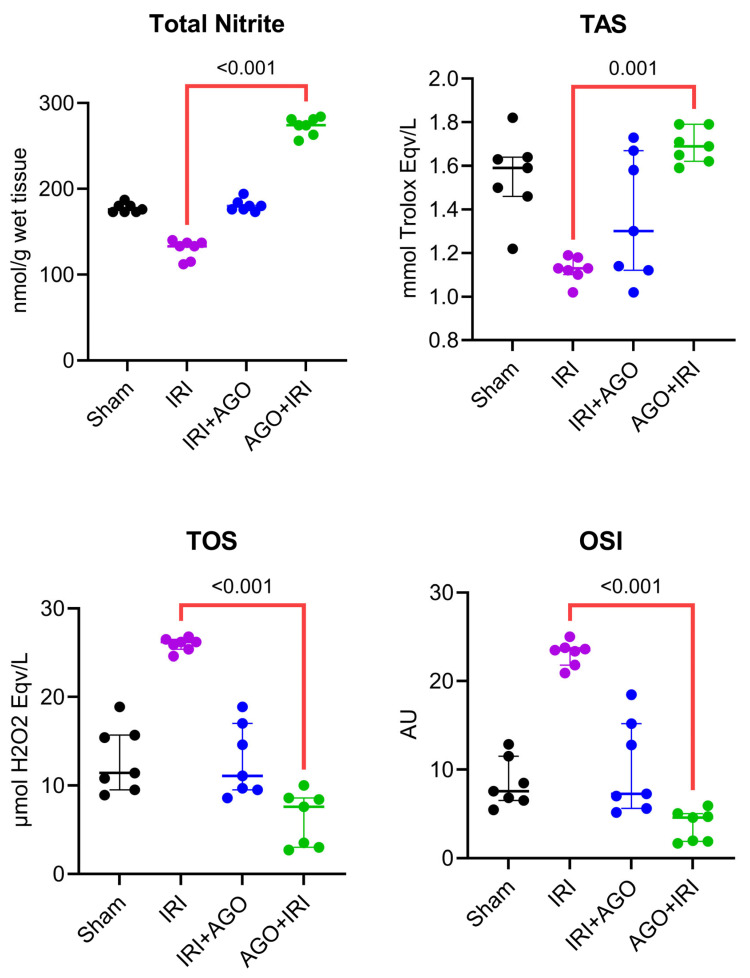
Individual data points of tissue NO-related and oxidative balance markers across experimental groups (Total nitrite, TAS, TOS, and OSI). Overall group differences were assessed by Kruskal–Wallis test, followed by Dunn’s multiple-comparisons test.

Oxidative Stress Index (OSI) values showed a significant difference among groups (H = 21.9, *p* < 0.001). Post-hoc analysis demonstrated that the IRI group differed significantly from the AGO+IRI group (*p* < 0.001), whereas the remaining pairwise comparisons were not statistically significant (adjusted *p* > 0.05). This finding indicates that OSI was most effectively suppressed in the AGO+IRI group. The detailed intergroup comparisons are presented in Table 1, and the corresponding tissue biochemical findings are illustrated in Figure 1 and Figure 2.

#### 2.1.2. AGO Modulates NO Metabolism, Attenuates Interleukin-6 (IL-6)–Associated Inflammatory Signaling, and Improves Biochemical Indicators of Liver Injury

Serum arginine levels differed significantly among the four groups (H = 8.63, *p* = 0.030). Dunn’s post-hoc analysis demonstrated a significant difference only between the IRI and AGO+IRI groups (adjusted *p* = 0.020), whereas all other pairwise comparisons were not significant (adjusted *p* > 0.05). Serum asymmetric dimethylarginine (ADMA) levels showed a significant overall difference among groups (H = 19.14, *p* < 0.001). Post-hoc analysis revealed that the IRI group differed significantly from both the IRI+AGO group (adjusted *p* = 0.030) and the AGO+IRI group (adjusted *p* < 0.001). No other pairwise comparisons reached statistical significance. Serum symmetric dimethylarginine (SDMA) levels differed significantly among groups (H = 15.44, *p* = 0.001). Dunn’s test identified significant differences between the Sham and IRI+AGO groups (adjusted *p* = 0.04) and between the IRI+AGO and AGO+IRI groups (adjusted *p* = 0.001). All remaining comparisons were not significant.

Serum tumor necrosis factor-alpha (TNF-α) levels were similar across all groups (H = 3.02, *p* = 0.390), with no significant post-hoc differences. Serum IL-1β levels did not significantly differ among groups (H = 7.70, *p* = 0.05), and no significant pairwise differences were identified in post-hoc analysis. Serum IL-6 levels differed significantly among groups (H = 14.71, *p* = 0.002). Dunn’s multiple comparisons test demonstrated that the IRI group was significantly different from the Sham group (adjusted *p*= 0.002) and from the IRI+AGO group (adjusted *p* = 0.030). No other pairwise comparisons reached statistical significance.

Serum lactate dehydrogenase (LDH) levels did not show significant intergroup differences (H = 3.18, *p* = 0.370), and no significant pairwise comparisons were detected. Serum aspartate aminotransferase (AST) levels did not differ significantly among groups (H = 3.99, *p* = 0.260), and no significant pairwise differences were observed (adjusted *p* > 0.05 for all). Serum alanine aminotransferase (ALT) levels were comparable across groups (H = 4.71, *p* = 0.190), with no significant differences detected in post-hoc analysis (adjusted *p* > 0.05 for all). Serum alkaline phosphatase (ALP) levels differed significantly among groups (H = 11.36, *p* = 0.010). Multiple comparisons test showed a significant difference between the Sham and IRI groups (adjusted *p* = 0.009), whereas other comparisons were not statistically significant. Serum gamma-glutamyl transferase (GGT) levels did not show a statistically significant difference among groups (H = 2.36, *p* = 0.500), and post-hoc analyis confirmed the absence of significant pairwise comparisons (adjusted *p* > 0.05 for all).

**Figure 3:**
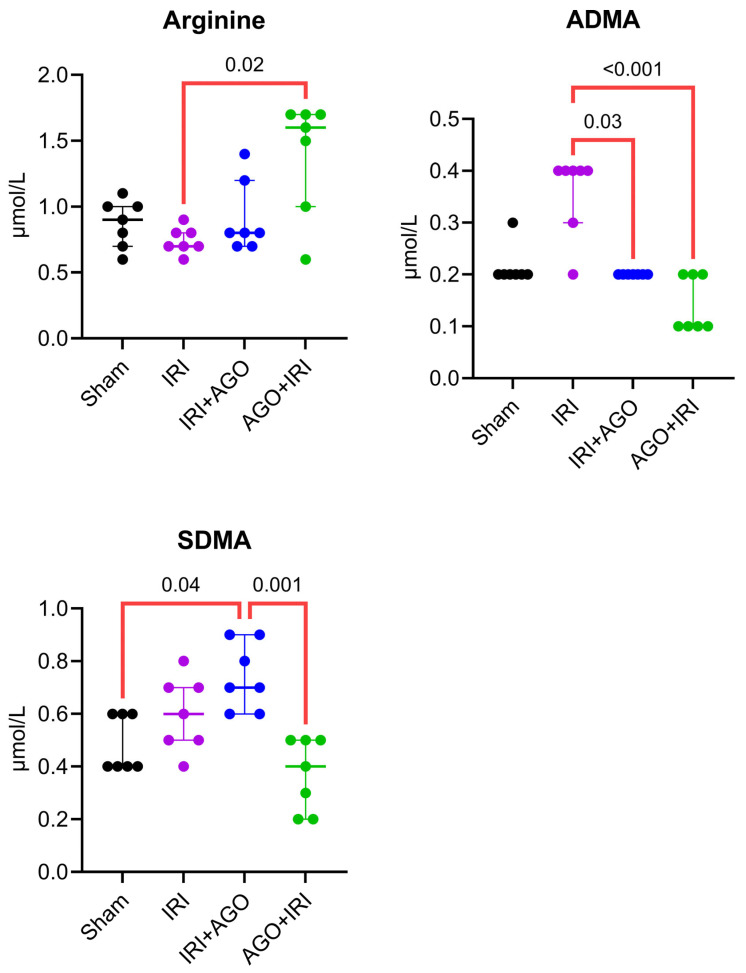
Individual data points of serum NO-related parameters across experimental groups (Arginine, ADMA, and SDMA). Overall group differences were assessed by Kruskal–Wallis test, followed by Dunn’s multiple-comparisons test.

**Figure 4:**
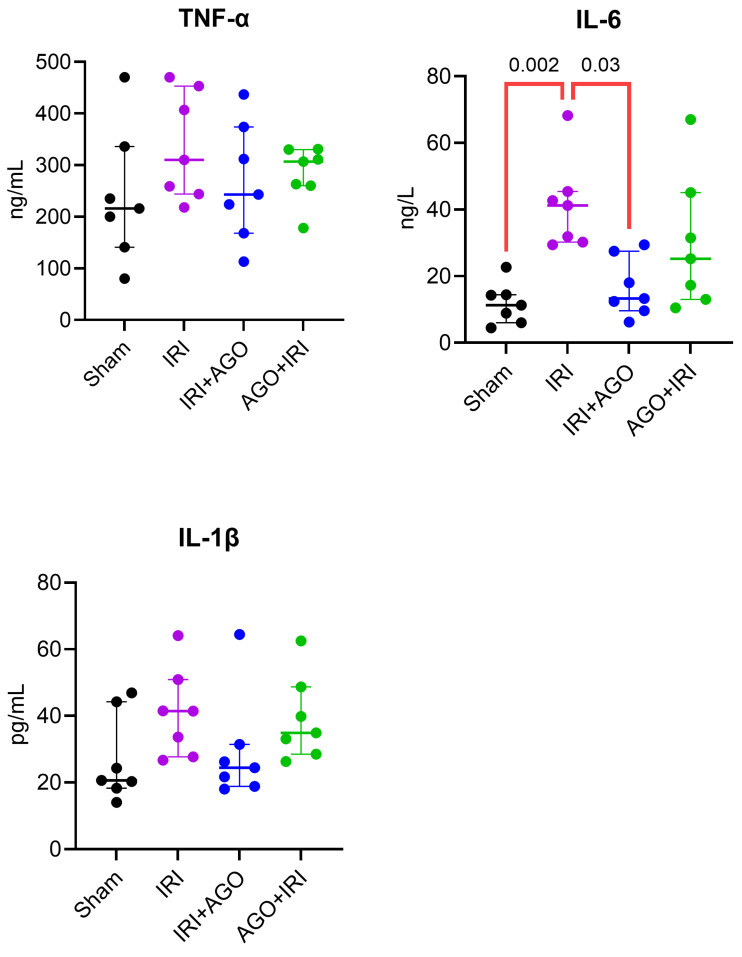
Individual data points of serum inflammatory markers across experimental groups (TNF-α, IL-1β, and IL-6). Overall group differences were assessed by Kruskal–Wallis test, followed by Dunn’s multiple-comparisons test.

**Figure 5:**
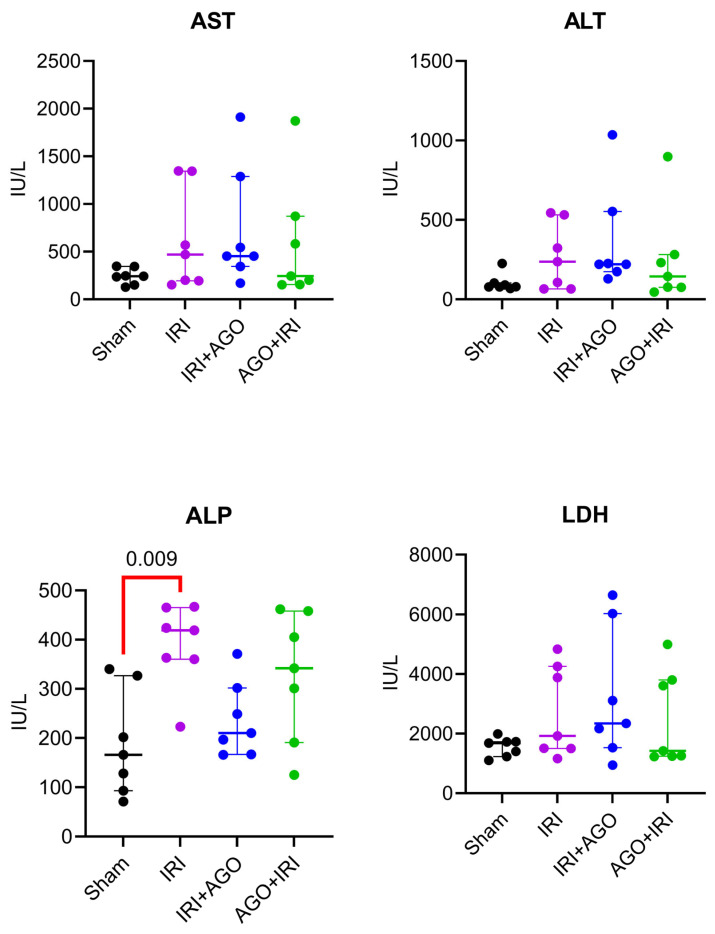
Individual data points of serum liver injury enzymes across experimental groups (AST, ALT, ALP, and LDH). Overall group differences were assessed by Kruskal–Wallis test, followed by Dunn’s multiple-comparisons test.

Collectively, significant serum alterations were primarily observed in NO-related parameters (arginine, ADMA, SDMA), IL-6, and ALP, whereas conventional liver enzymes (AST, ALT, LDH) and the cytokines TNF-α and IL-1β did not demonstrate statistically significant intergroup differences. The detailed intergroup comparisons are presented in Table 2, and the corresponding serum biochemical findings are illustrated in Figure 3, Figure 4 and Figure 5.

### 2.2. AGO Reduces Hepatocellular Apoptosis and Histopathological Injury

**Figure 6:**
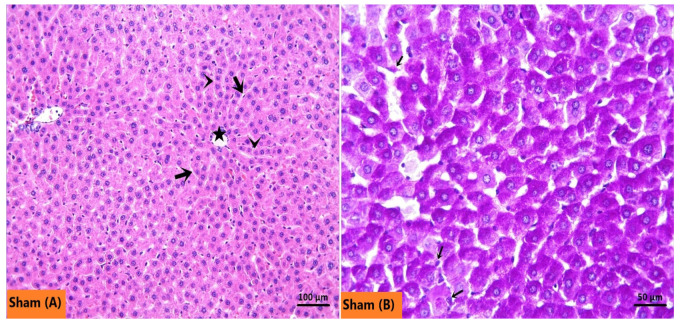
Representative micrographs showing the Sham group liver. (**A**) In the H&E-stained sections, the liver tissue in the Sham group shows a normal histological architecture, with hepatocyte cords (arrowheads) and sinusoids (thick arrows) around the central vein (asteriks). (**B**) In the PAS-stained sections, glycogen content of hepatocytes and Kupffer cells (thin arrows) appeared prominently in purple-pink.

**Figure 7:**
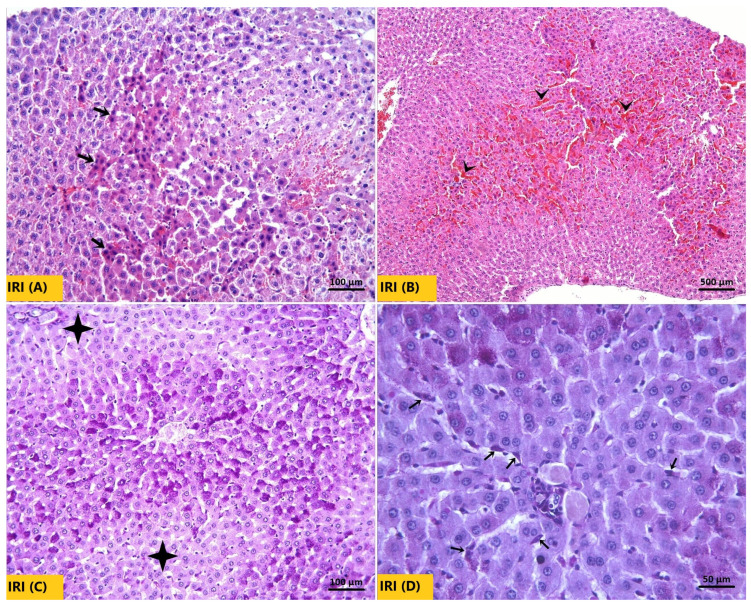
Representative micrographs showing the IRI group liver. (**A**) In the H&E-stained sections, numerous necrotic hepatocytes (thick arrows) characterized by strongly eosinophilic cytoplasm, intensely stained nuclei, and shrinkage are prominent. (**B**) In the H&E-stained sections, severe sinusoidal congestion (arrowheads) is observed. (**C**) In the PAS-stained sections, the majority of hepatocytes appeared paler purple-pink (four-pointed star), indicating a decrease in glycogen content. (**D**) In the PAS-stained sections, purple-pink-stained Kupffer cells (thin arrows) are observed.

**Figure 8:**
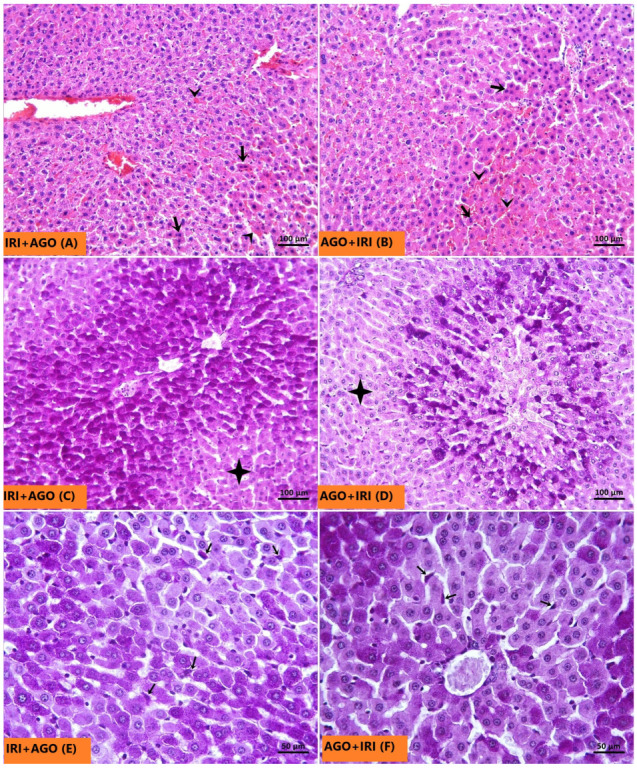
In the H&E-stained sections, a marked reduction in the density of necrotic hepatocytes (thick arrows) and sinusoidal congestion is observed in the IRI+AGO (**A**) and AGO+IRI (**B**) groups compared with the IRI group, sinusoidal congestion (arrowheads). In the PAS-stained sections, a higher glycogen content (four-pointed star) is exhibited by hepatocytes in the IRI+AGO group (**C**) compared with those in the AGO+ IRI group (**D**). In the AGO+ IRI group, a marked decrease in glycogen content is observed in hepatocytes surrounding the central vein (**D**). In the PAS-stained sections, purple-pink Kupffer cells (thin arrows) are observed in the IRI+AGO (**E**) and AGO+IRI (**F**) groups.

The Sham group, as seen in Figure 6A, exhibited normal hepatocyte architecture. The glycogen-rich hepatocyte cytoplasm and Kupffer cells showed prominent pink-purple staining with PAS (Figure 6B). In the IRI group, H&E-stained sections demonstrated a marked increase in necrotic hepatocytes characterized by strongly eosinophilic cytoplasm, hyperchromatic and shrunken nuclei (Figure 7A). Sinusoidal congestion was also a prominent feature (Figure 7B). Consistent with these findings, PAS staining was weaker, indicating reduced glycogen accumulation in hepatocytes (Figure 7C). The number of Kupffer cells in the IRI group did not differ significantly from that in the Sham group (Figure 7D). In both AGO regimen groups, necrotic hepatocyte density and sinusoidal congestion were substantially reduced compared with the IRI group (Figure 8A,B). Notably, pre-ischemic AGO (AGO+IRI) administration was more effective than at the onset of reperfusion treatment (IRI+AGO) in preserving hepatocellular glycogen stores (Figure 8C,D). The highest number of Kupffer cells was observed in the IRI+AGO group; however, there was no statistically significant difference between the IRI and AGO+IRI groups (Figure 8E,F). Scores for sinusoidal congestion, necrotic hepatocytes, glycogen loss, and Kupffer cell counts for all groups are presented in Table 3.

### 2.3. AGO Administration Attenuates Caspase-3 Activation

**Figure 9:**
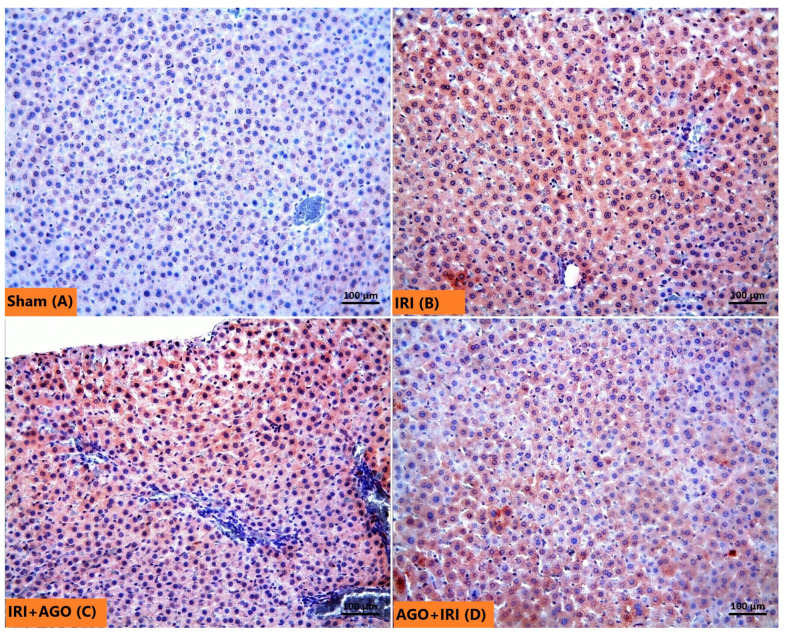
Representative micrographs showing caspase-3 labeling (brown coloration) of the liver from different experimental groups. (**A**) Sham group shows trivial labeling in hepatocytes. (**B**) IRI group shows caspase-3 labeling of hepatocytes. (**C**) IRI+AGO group shows caspase-3 labeling, which is similar to the IRI group. (**D**) AGO+IRI group shows somewhat diminished staining compared to the IRI group. In all panels, nuclei are counterstained with hematoxylin.

In the Sham group, caspase-3 immunolabeling was minimal, with only occasional hepatocytes exhibiting weak staining (Figure 9A). In contrast, the IRI group demonstrated markedly increased caspase-3-positive hepatocytes, indicating pronounced apoptotic activity (Figure 9B). Administration of AGO at the onset of reperfusion resulted in limited attenuation of caspase-3 expression compared with the IRI group (Figure 9C). However, pre-ischemic AGO treatment resulted in markedly lower caspase-3 labeling, indicating a more pronounced attenuation of apoptotic activity when the drug was administered before the ischemic insult (Figure 9D). Quantitative densities of caspase-3-positive cells for all groups are presented in Table 3.

### 2.4. Integrated Mechanistic Interpretation of AGO-Mediated Hepatoprotection

Taken together, the present findings suggest a coordinated pathophysiological pattern underlying hepatic IRI. Hepatic IRI was characterized by a marked increase in oxidative stress markers (TBARS, TOS, and OSI), accompanied by depletion of endogenous antioxidant defenses (GSH, CAT, and GSHPx). These alterations were also associated with disturbances in NO parameters (arginine, ADMA, and SDMA) and increased levels of the pro-inflammatory cytokine IL-6, suggesting that oxidative stress, NO dysregulation, and inflammatory signaling may represent interconnected components of hepatic IRI.

Administration of AGO attenuated many of these alterations, particularly when administered before ischemia (AGO+IRI). This intervention was associated with reduced oxidative stress markers, partial restoration of antioxidant enzyme activity, and attenuation of early injury-associated cytokine signaling (particularly IL-6). Collectively, these findings suggest that AGO may exert hepatoprotective effects through coordinated modulation of oxidative stress, NO metabolism, and inflammatory signaling pathways.

## 3. Discussion

Hepatic IRI is a well-recognized consequence of temporary interruption of hepatic blood flow during hepatobiliary surgery and LT [53]. Although restoration of perfusion is essential to re-establish metabolic homeostasis, reperfusion paradoxically aggravates cellular injury through oxidative stress, inflammatory activation, microcirculatory impairment, and apoptotic signaling [54,55]. Clinically, the extent of injury is generally estimated using routine biochemical markers such as AST, ALT, LDH, and GGT, although these parameters often reflect damage only after substantial hepatocellular disruption has occurred [56].

In the present study, a total warm hepatic ischemia–reperfusion model produced a consistent pattern of oxidative and inflammatory tissue injury. Lipid peroxidation and oxidant burden increased markedly (TBARS, TOS, OSI), whereas endogenous antioxidant defenses (GSH, CAT, and GSHPx) were depleted. AGO administration attenuated these alterations, and the protective effect was more pronounced when AGO was administered before ischemia (AGO+IRI) than when given at the onset of reperfusion (IRI+AGO). This consistent pattern across multiple redox parameters suggests that pharmacological modulation prior to reperfusion may limit the oxidative burst that characterizes early hepatic IRI [57,58].

ROS play a central role in the pathogenesis of hepatic IRI by overwhelming endogenous antioxidant defenses and triggering lipid peroxidation, mitochondrial dysfunction, and hepatocellular injury [59,60,61,62,63,64,65,66]. In our model, this mechanism was reflected by elevated TBARS, TOS, and OSI together with decreased GSH, CAT, and GSHPx in the IRI group. Restoration of these parameters in AGO-treated animals therefore indicates that AGO mitigates oxidative injury primarily by supporting endogenous antioxidant capacity.

Microcirculatory dysfunction represents another critical component of hepatic IRI. Sinusoidal congestion and Kupffer cell activation contribute to inflammatory signaling and impair sinusoidal perfusion, processes closely linked to NO bioavailability and endothelial function [67]. Reduced NO bioavailability promotes vasoconstriction and exacerbates hepatocellular injury through endothelial dysfunction and inflammatory amplification. In the present study, CAT and GSHPx activities were markedly reduced following IRI but significantly restored in AGO-treated groups, particularly in animals receiving pre-ischemic AGO administration. These findings suggest that AGO enhances enzymatic antioxidant defenses and may indirectly improve microcirculatory stability during reperfusion.

Alterations in NO-related parameters further support this interpretation. Depletion of NADPH and reduced L-arginine availability during IRI can limit endothelial NO synthesis and impair sinusoidal perfusion [32]. Consistent with this mechanism, the present study demonstrated decreased L-arginine and nitrite levels in the IRI group, whereas AGO administration partially restored these parameters. Such changes suggest improved endothelial signaling and microvascular function during early reperfusion.

Inflammatory processes are known to contribute to the progression of hepatic IRI. Activation of Kupffer cells and recruitment of inflammatory cells during reperfusion can promote the release of cytokines such as TNF-α, IL-1β, and IL-6, which interact with oxidative stress pathways and may enhance hepatocellular injury and apoptotic signaling [68,69,70]. However, in the present experimental model evaluated 1 h after reperfusion, a full inflammatory cytokine response was not observed. Among the measured cytokines, only IL-6 levels increased significantly following IRI, whereas TNF-α and IL-1β did not show statistically significant changes compared with the Sham group. Accordingly, the cytokine pattern observed in this study likely reflects early reperfusion-related signaling rather than a fully developed inflammatory cascade. Notably, AGO treatment was associated with a reduction in IL-6 levels, particularly in the pre-ischemic administration group. This finding suggests that AGO may modulate early injury-associated cytokine signaling during the initial phase of reperfusion, rather than broadly suppressing the entire inflammatory response.

Endogenous antioxidant enzyme systems represent a major protective mechanism against oxidative injury during IRI [71,72,73,74,75]. In our study, CAT activity decreased markedly in the IRI group, indicating impairment of antioxidant defenses, whereas AGO administration restored CAT activity, particularly in the AGO+IRI group. Similarly, GSHPx activity—which declined following IRI—was significantly improved in AGO-treated animals. These findings support the concept that AGO contributes to hepatocellular protection by restoring enzymatic antioxidant capacity rather than by targeting a single downstream oxidative marker.

Evaluation of global redox status further supported this interpretation. TAS levels decreased and TOS levels increased after IRI, indicating a marked shift toward oxidative imbalance [76,77]. AGO administration reversed this pattern, producing higher TAS and lower TOS values compared with untreated IRI animals. These results indicate that AGO improves systemic redox homeostasis in addition to restoring individual antioxidant enzymes.

Oxidative stress and inflammation are closely interconnected through redox-sensitive signaling pathways such as nuclear factor-κB (NF-κB), which regulates the transcription of pro-inflammatory cytokines including IL-1, IL-6, and TNF-α [78,79]. Antioxidant interventions may attenuate cytokine synthesis by limiting ROS-driven NF-κB activation. In the present study, AGO administration was associated with a clear reduction in IL-6 and a non-significant decrease in IL-1β, whereas TNF-α levels remained unchanged. This differentiated response suggests that AGO modulates inflammatory signaling selectively rather than uniformly suppressing all cytokine pathways.

Interpretation of conventional liver enzyme results requires consideration of reperfusion timing. Tissue-level oxidative and apoptotic changes occur rapidly during early reperfusion, whereas serum biochemical markers may rise later due to delayed enzyme release into the circulation [27,44,80]. Because AST and ALT have relatively long plasma half-lives, early experimental observation periods may not fully capture biochemical recovery despite clear histopathological improvement. This temporal dissociation likely explains why tissue-level protection was evident in the present study even though serum enzyme changes were limited.

**Figure 10:**
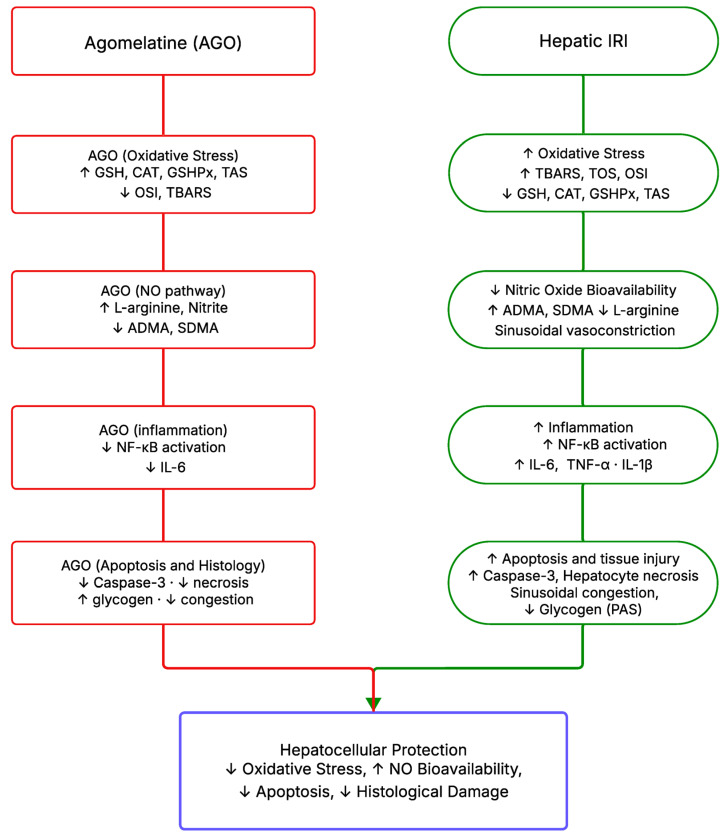
Mechanisms underlying the cytoprotective effects of AGO in hepatic IRI. Based on the findings of the present study and previous experimental evidence, AGO attenuates oxidative stress, improves NO bioavailability, modulates early inflammatory signaling, and reduces apoptosis and histological liver injury [49,50,83].

AGO, originally developed as an atypical antidepressant acting through melatonin MT1/MT2 receptor agonism and 5-HT2C receptor antagonism [81,82], has been shown in several experimental studies to exert antioxidant properties in various tissues, including the liver [40,83]. The present findings suggest that its cytoprotective properties extend beyond its established central nervous system actions. In the current study, IRI-induced lipid peroxidation and oxidative imbalance—reflected by elevated TBARS, TOS, and OSI together with depleted GSH, CAT, GSHPx, and TAS levels—were significantly attenuated following AGO administration. These findings are consistent with previously reported free-radical scavenging and antioxidant enzyme-restoring effects of AGO in hepatic and renal IRI models. In addition, AGO modulated nitric-oxide-related parameters. ADMA, a potent endogenous inhibitor of endothelial NO synthesis, was significantly elevated in the IRI group and was reduced by both AGO regimens, suggesting partial restoration of NO bioavailability. Furthermore, L-arginine and nitrite concentrations increased markedly, with the highest values observed in the AGO+IRI group. SDMA displayed a distinct pattern: it was elevated in both the IRI and IRI-AGO groups compared with the Sham group but tended to return toward Sham levels in the IRI-AGO group relative to the AGO-IRI group, the mechanistic relevance of which requires further investigation. At the inflammatory level, AGO significantly attenuated IL-6 elevation. Although TNF-α and IL-1β did not reach statistical significance in this model—likely reflecting the early reperfusion time point of 60 min rather than a fully developed inflammatory cascade—the selective modulation of IL-6 by AGO is consistent with antioxidant-mediated suppression of redox-sensitive inflammatory signaling pathways, including NF-κB, as reported in previous studies [49,83,84]. Histopathological and immunohistochemical analyses further supported these biochemical observations. AGO treatment reduced necrotic hepatocyte density, sinusoidal congestion, and caspase-3 expression. Notably, attenuation of apoptotic activity was more pronounced in the pre-ischemic group (AGO+IRI), whereas glycogen preservation appeared more evident in the IRI-AGO group, suggesting that the timing of AGO administration may influence different aspects of hepatocellular protection. Collectively, these findings indicate that AGO attenuates early reperfusion injury through coordinated modulation of oxidative stress, NO metabolism, and inflammatory signaling, with pre-ischemic administration providing broader and more consistent protection across biochemical and apoptotic parameters. The mechanisms underlying the cytoprotective effects of AGO in hepatic ischemic injury, based on the findings of the present study and previous experimental studies on AGO, are summarized schematically in Figure 10.

From a clinical perspective, the experimental condition most closely corresponding to hepatic IRI is LT or major hepatic resection. In transplantation, IRI contributes to complications such as EAD and primary graft dysfunction, which are typically monitored using routine biochemical markers such as AST, ALT, bilirubin, and INR during the early postoperative period [6,20,85,86]. Because these parameters may detect injury relatively late, transplant research increasingly emphasizes earlier indicators of graft perfusion and cellular injury together with mechanistic tissue-level assessment. Various pharmacologic approaches—including antioxidants such as melatonin and N-acetylcysteine—have been explored to attenuate IRI in this setting.

Within this context, the present findings suggest that AGO may exert experimental hepatoprotective effects by limiting early oxidative and inflammatory injury during reperfusion. However, any translational interpretation must be approached with caution. AGO has a recognized hepatic safety signal in clinical use, including cases of clinically significant hepatotoxicity [46,51,87,88] Consequently, the present results should be regarded primarily as mechanistic experimental evidence rather than as support for direct clinical application.

This study has several limitations that should be considered. First, the relatively small sample size, although consistent with ethical principles in experimental animal research and comparable to many hepatic IRI studies, may limit statistical power for variables with high biological variability, particularly serum transaminases such as AST and ALT. However, post hoc power analysis indicated adequate power to detect differences in oxidative stress, antioxidant, inflammatory, and histopathological parameters. Second, liver tissue sampling was performed 1 h after the onset of reperfusion, representing a very early time point in the evolution of hepatic IRI. The early sampling time may also explain why conventional serum liver enzymes such as AST, ALT, and LDH did not show statistically significant differences between the Sham and IRI groups, despite the presence of clear oxidative and tissue-level alterations. This short interval may partly explain the moderate histological changes and the temporal dynamics of inflammatory markers observed in the study and therefore constitutes an important limitation of the experimental model. Nevertheless, numerous studies investigating antioxidant or cytoprotective agents in hepatic IRI employ ischemia durations of approximately 30–60 min followed by early reperfusion periods ranging from 1 to several hours, which are sufficient to induce measurable oxidative stress, inflammatory responses, and apoptotic signaling. Third, apoptosis was evaluated only by caspase-3 immunohistochemistry. Although caspase-3 is a key executioner marker, additional assays such as Terminal deoxynucleotidyl transferase–mediated dUTP nick end labeling (TUNEL), Annexin V, or Poly(ADP-ribose) polymerase (PARP) cleavage could provide a more comprehensive assessment of apoptotic pathways. Fourth, AGO was administered at a moderate dose for a short duration in an acute injury model. Longer treatment periods and extended observation times may better clarify sustained protective mechanisms. Finally, the study did not include a positive control using a well-established hepatoprotective agent such as melatonin or N-acetylcysteine, which would allow comparison with established antioxidant strategies. Despite these limitations, the findings indicate that AGO attenuates hepatic IRI through antioxidant, anti-inflammatory, and anti-apoptotic effects. The protection was more pronounced when AGO was administered before ischemia, suggesting that pre-ischemic modulation of oxidative and inflammatory pathways may limit early reperfusion injury.

## 4. Materials and Methods

### 4.1. Animals and Ethical Approval

Twenty-eight Sprague Dawley rats, 11–12 weeks of age and weighing 230–300 g, were obtained from the Inonu University Experimental Animal Research Center (Malatya, Türkiye). The rats were housed under standard laboratory conditions (21 ± 2 °C, 60 ± 5% humidity, and a 12:12 h light–dark cycle) with ad libitum access to standard chow and water. All procedures were performed in accordance with the NIH Guide for the Care and Use of Laboratory Animals [89]. Ethical approval was obtained from the Inonu University Faculty of Medicine Animal Experiments Ethics Committee (Approval No. 2016/A-36, 07 March 2016). This experimental study was supported by the Inonu University Scientific Research Projects Coordination Unit (Project No: 2016/169).

### 4.2. Experimental Design

Twenty-eight rats were randomly allocated into four equal groups (*n* = 7 per group): Sham (no IRI), IRI group, IRI+AGO group (AGO 20 mg/kg administered immediately at the onset of reperfusion), and AGO+IRI group (AGO 20 mg/kg administered 1 h before the beginning of ischemia). In the IRI+AGO group, AGO was administered by oral gavage immediately after removal of the vascular clamp and confirmation of reperfusion, while the animals were still under anesthesia and before abdominal closure. To minimize the risk of aspiration, the heads of the anesthetized rats were kept in an upright position during and immediately after gavage.

The AGO dose was selected based on the study by Karakus et al. [50]. Valdoxan^®^ 25 mg film-coated tablets (Servier, Istanbul, Turkey) were powdered, and the required amount of powder was suspended in distilled water. A final volume of 1 mL was administered to each rat by oral gavage, with the dose adjusted according to body weight. The surgical procedure used in this study was performed as previously described [90]. Rats were anesthetized with intraperitoneal ketamine (75 mg/kg; Ketasol 10% *w*/*v* injectable solution; Richter Pharma AG, Wels, Austria) and xylazine (8 mg/kg; XylazinBio 2% injectable solution; Bioveta PLC, Ivanovice na Hané, Czech Republic). The abdominal region was shaved and disinfected with 10% povidone–iodine (Batticon antiseptic solution, ADEKA Pharmaceuticals, İstanbul, Türkiye), and a midline laparotomy with minimal dissection was performed to expose the abdominal organs. In the Sham group, only laparotomy and abdominal closure were performed without vascular occlusion. In the IRI, IRI+AGO, and AGO+IRI groups, a total warm hepatic ischemia–reperfusion model was used, in which ischemia was induced by 1 h by occluding the hepatic artery, portal vein, and bile duct with a non-traumatic microvascular clamp. The liver was then reperfused for 1 h after removal of the clamp. After declamping, restoration of hepatic blood flow was visually confirmed before closure of the incision. During surgery, body temperature was maintained at approximately 37.5 °C using a heating lamp. Fluid loss was replaced by intraperitoneal injection of 3 mL of warm (37 °C) saline before abdominal closure. At the end of the reperfusion period, all rats were euthanized with an overdose of ketamine (225 mg/kg) and xylazine (24 mg/kg). Blood samples were collected from the inferior vena cava immediately before sacrifice for biochemical analysis. Hepatectomy was then performed, and a portion of the liver tissue was fixed in 10% neutral buffered formalin for histopathological analysis, while the remaining tissue was stored at −70 °C for biochemical analysis.

### 4.3. Biochemical Analyses

Blood samples were stored at −70 °C until biochemical testing. Liver tissue samples were homogenized in cold phosphate buffer (20 mmol, pH 7.4) containing a protease inhibitor cocktail using an Ultra-Turrax T25 basic homogenizer (IKA, Istanbul, Türkiye) at 16,000 rpm for 3 min at +4 °C. TBARS analysis was performed directly on the homogenates. The remaining homogenates were centrifuged at 10,000× *g* for 20 min at +4 °C, and the resulting supernatants were used for the measurement of GSH, GSHPx, CAT, total nitrite, TOS, TAS, OSI, IL-1β, IL-6, and TNF-α. Blood samples were centrifuged at 2000× *g* for 10 min at +4 °C, and the resulting serum samples were used to determine GSH, TAS, TOS, OSI, total nitrite, L-arginine, ADMA and SDMA.

#### 4.3.1. TBARS Measurement

Tissue TBARS levels were determined according to the method of Ohkawa et al. [91]. In this assay, TBARS reacts with thiobarbituric acid (TBA) under acidic and high-temperature conditions to form a reddish-pink, the absorbance of which is measured at 532 nm using a Synergy H1 microplate reader (BioTek, Santa Clara, CA, USA). The intensity of the chromogen is directly proportional to the TBARS concentration in the sample. Tissue TBARS content was expressed as nmol/g wet tissue.

#### 4.3.2. GSH Measurement

GSH levels in tissue samples were measured according to the method of Ellman [92]. In this assay, GSH reacts with 5,5′-dithiobis-2-nitrobenzoic acid (DTNB) the absorbance of which was recorded at 410 nm using a Synergy H1 microplate reader (Agilent Technologies, Santa Clara, CA, USA). The intensity of the color is directly proportional to the concentration of GSH in the sample. Tissue GSH levels were expressed as nmol/g wet tissue.

#### 4.3.3. CAT Activity Measurement

CAT activity levels of tissue samples were measured according to the method of Aebi [93]. In this assay, CAT catalyzes the decomposition of hydrogen peroxide (H_2_O_2_) into water and oxygen, and the resulting decrease in absorbance at 240 nm is monitored using a Synergy H1 microplate reader. The rate of absorbance reduction is directly proportional to CAT activity in the sample. Tissue CAT activity levels were expressed as U/g protein.

#### 4.3.4. GSHPx Activity Measurement

GSHPx activity in tissue samples was measured according to the method of Paglia and Valentine [94]. GSHPx catalyzes the reduction of H_2_O_2_ to water using GSH as an electron donor, during which GSH is converted to oxidized glutathione (GSSG). In the presence of glutathione reductase and nicotinamide adenine dinucleotide phosphate (NADPH), GSSG is reconverted to GSH, while NADPH is oxidized to NADP^+^. NADPH exhibits maximal absorbance at 340 nm, and as it is oxidized during the reaction, a progressive decrease in absorbance at 340 nm occurs. This decrease was monitored using a Synergy H1 microplate reader and is directly proportional to GSHPx enzymatic activity in the sample. Tissue GSHPx activity levels were expressed as U/g protein.

#### 4.3.5. NO Measurement

As total nitrite levels are widely accepted as an index of endogenous NO production [95,96], NO levels in serum and liver tissue supernatants were evaluated by measuring total nitrite concentrations. The assessment was performed according to the method described by Ozbek et al. [97]. The absorbance of the resulting azo compound was measured at 548 nm using a Synergy H1 microplate reader, and nitrite concentrations were calculated using standard calibration curves. Total nitrite levels in tissue supernatants were expressed as nmol/g wet tissue.

#### 4.3.6. Measurement of IL-1β, IL-6, TNF-α Levels

IL-1β, IL-6, and TNF-α levels in liver tissue supernatants were measured using Elabscience/RayBiotech (Houston, TX, USA, Catalog no: ELR-IL6, Catalog no: ELR-Tnf a, Catalog no: ELR-IL 1b) proprietary ELISA kits, following the manufacturer’s instructions.

#### 4.3.7. Measurement of Serum ADMA, SDMA and L-Arginine Levels

Serum ADMA, SDMA, and L-arginine concentrations were measured using high-performance liquid chromatography (HPLC) with Eureka (Chiaravalle, Italy, Catalog no: Z58010) proprietary kits, following the manufacturer’s instructions. Results were expressed as µmol/L, providing a standardized quantitative comparison across samples.

#### 4.3.8. Measurement of Supernatant and Serum TAS Levels

The TAS levels of serum and tissue supernatants were measured using a Rel Assay Diagnostics (Gaziantep, Türkiye, Catalog No: RL0017) proprietary kit, following the manufacturer’s instructions. The kit instructions suggest mixing 500 μL reagent 1 (measurement buffer) and 30 μL supernatant and measuring the absorbance at 660 nm with a microplate reader. TAS was measured at 25 °C with the BioTek Synergy H1 device (BioTek Instruments, Inc., Winooski, VT, USA). 25 μL reagent 2 (colored ABTS solution) was added to the solution and it was incubated for 10 min. The absorbance was recorded at 660 nm to determine the TAS. The water-soluble vitamin E compound, trolox was used as the calibrator. TAS levels in both serum and supernatant samples were expressed as mmol Trolox equivalent/L, providing a standardized index of overall antioxidant capacity.

#### 4.3.9. Measurement of Supernatant and Serum TOS Levels

TOS levels in serum and tissue supernatants were measured using a Rel Assay Diagnostics (Gaziantep, Türkiye, Catalog No: RL0017) proprietary kit, following the manufacturer’s instructions. Ferric ions and the chromogenic solution create a colored compound. Following the kit instructions, 500 μL reagent 1 (measurement buffer) and 75 μL supernatant were combined, and a microplate reader was utilized to measure the absorbance at 660 nm. TOS was determined with a BioTek Synergy H1 device at 25 °C (BioTek Instruments, Inc., Winooski, VT, USA). 25 μL reagent 2 (colored ABTS solution) was added to the mixture, and the outcome was incubated for 10 min. The absorbance was observed at 530 nm to determine the TAS⋅H_2_O_2_ was employed as the calibrator. TOS values were expressed as μmol H_2_O_2_ equivalent/L, providing a quantitative index of total oxidant burden in the samples.

#### 4.3.10. OSI Estimation

The OSI was calculated using the formula OSI = TOS (µmol H_2_O_2_ equivalent/L)/TAS (mmol Trolox equivalent/L), as described by Erel in 2005 [77].

#### 4.3.11. Measurement of Routine Liver Function Tests

Blood samples were centrifuged to obtain serum, and biochemical parameters were analyzed in the central clinical biochemistry laboratory of Inonu University Turgut Özal Medical Center using an automated analyzer (Architect c16000, Abbott Diagnostics, Abbott Park, IL, USA). The reference intervals used by the laboratory were as follows: AST 0–40 U/L, ALT 0–41 U/L, ALP 40–130 U/L, GGT 9–64 U/L, and LDH 0–248 U/L. These reference ranges correspond to those routinely applied in the laboratory where the analyses were performed.

### 4.4. Histological and Histochemical Analyses

Liver tissue was fixed in 10% neutral buffered formalin, routinely processed, and embedded in paraffin. Sections cut at 4 μm were mounted on slides and stained with hematoxylin and eosin (H&E) for general histological assessment. Additional sections were stained using the periodic acid–Schiff (PAS) method to evaluate glycogen accumulation in hepatocytes and Kupffer cells. Liver injury was assessed semiquantitatively, based on the presence and extent of hepatocellular necrosis, sinusoidal congestion, and loss of glycogen deposits. A 3-point histopathological scoring system was used: 0 = normal liver, 1 = mild damage involving up to 25% of the liver, 2 = moderate damage involving 25–50%, and 3 = severe damage affecting ≥50% of hepatic tissue [24,98]. For each rat, ten fields were examined at 20× magnification. Kupffer cells were quantified manually using the Leica Q Win digital imaging system (Leica Microsystems Imaging Solutions Ltd., Cambridge, UK), with three sections evaluated per animal. In each section, ten randomly selected fields at 40× magnification were analyzed, providing a total of 30 evaluated fields per rat. Ten fields were randomly selected in each section at 40× magnification, so a total of 30 sites were examined for each rat.

### 4.5. Immunohistochemical Analysis

Sections were cut from paraffin blocks at 4 μm, dewaxed, rehydrated through a graded alcohol series, and processed for immunohistochemistry. For antigen retrieval, sections were placed in pH 6.0 citrate buffer and heated in a pressure cooker at 95–100 °C for 20 min, followed by cooling at room temperature for 20 min and washing with phosphate-buffered saline (PBS). Endogenous peroxidase activity was blocked using 3% H_2_O_2_ solution (TA-125-HP, Thermo Fisher Scientific, Fremont, CA, USA) for 15 min at room temperature. After rinsing with PBS, sections were incubated with a protein block solution (TP-125-HL, Thermo Fisher Scientific, Fremont, CA, USA). Sections were then incubated for 60 min with rabbit polyclonal anti-caspase-3 primary antibody (RB-1197-P, Thermo Fisher Scientific, Fremont, CA, USA). This step was followed by a 20-min incubation at room temperature with goat anti-polyvalent secondary antibody (TP-125-HL, Thermo Fisher Scientific, Fremont, CA, USA). After washing with PBS, sections were incubated for 20 min with streptavidin–peroxidase. Chromogenic visualization was performed using AEC (3-amino-9-ethylcarbazole) chromogen (TA-125-HA, Thermo Fisher Scientific, Fremont, CA, USA). One drop of chromogen was diluted in 1 mL of substrate solution, and sections were incubated for 10 min. Slides were counterstained with Mayer’s hematoxylin for 1 min, rinsed with tap water, and air-dried. Caspase-3-positive cells showed brown cytoplasmic labeling. Immunohistochemical expression was scored semiquantitatively as follows: 0 = no expression, 1 = minimal expression (≤25% of the liver), 2 = moderate expression (25–50%), and 3 = strong expression (≥50%). For each rat, ten fields were examined at 20× magnification. All sections were evaluated using a Leica DFC280 light microscope and the Leica Q Win Image Analysis System (Leica Micros Imaging Solutions Ltd., Cambridge, UK).

### 4.6. Statistical Analysis

All statistical analyses were performed using IBM SPSS Statistics (IBM Corp., Armonk, NY, USA) and GraphPad Prism version 10.6.1 for Windows (GraphPad Software, Boston, MA, USA). SPSS version 22.0 was used for descriptive statistics and for the analysis of histopathological data, whereas SPSS version 25.0 and GraphPad Prism was used for the statistical analysis and graphical presentation of biochemical parameters. Histopathological scores were compared using the Kruskal–Wallis test for overall group comparisons, followed by Conover’s multiple-comparisons test for post-hoc analysis. Biochemical parameters were analyzed using the Kruskal–Wallis test for overall group comparisons. When a significant difference was detected, Dunn’s multiple-comparisons test with Bonferroni correction was applied for pairwise comparisons. In accordance with the non-parametric statistical approach, biochemical results are presented as median with interquartile range (Q1–Q3), while histopathological findings are expressed as median with minimum–maximum values. A *p* value < 0.05 was considered statistically significant.

## 5. Conclusions

The present study demonstrates that AGO exerts cytoprotective effects in experimental hepatic IRI. Both pre-ischemic (prophylactic) administration and therapeutic administration at the onset of reperfusion attenuated biochemical, histopathological, and immunohistochemical indicators of liver injury. However, the protective effect was more pronounced when AGO was administered pre-ischemic period. This was evidenced by lower TBARS and OSI levels, better preservation of antioxidant defenses including GSH, CAT, and GSHPx, reduced hepatocellular necrosis and sinusoidal congestion, improved glycogen retention, and decreased caspase-3 expression. These findings indicate that AGO primarily limits oxidative stress-related tissue injury and apoptotic signaling associated with hepatic reperfusion rather than fully reversing established damage. The partial reduction in inflammatory mediators further suggests that modulation of oxidative stress may represent a central mechanism underlying its hepatoprotective action. Collectively, the results suggest that AGO may represent a promising experimental pharmacological strategy for attenuating the oxidative and apoptotic components of hepatic IRI. Further studies incorporating longer reperfusion periods and detailed mechanistic analyses are required to clarify its potential translational relevance.

## Figures and Tables

**Table 1:** Biochemical parameters measured in the liver tissue of the different experimental groups.

Parameters	Sham	IRI	IRI-AGO	AGO-IRI	*p*
TBARS	583 (570–600)	1095 (1079–1119)	519 (476–600)	459 (419–499)	<0.001
GSH	843 (712–907)	500 (494–542)	776 (760–984)	1471 (1292–1519)	<0.001
CAT	53 (51–65)	36 (30–38)	55 (49–62)	73 (72–74)	<0.001
GSHPx	121 (115–152)	77 (73–87)	155 (121–173)	171 (160–198)	<0.001
Total Nitrite	176 (173–180)	133 (115–137)	180 (176–184)	274 (263–281)	<0.001
TAS	1.6 (1.5–1.6)	1.1 (1.1–1.2)	1.3 (1.1–1.7)	1.7 (1.6–1.8)	0.002
TOS	11.4 (9.5–15.7)	26 (25–27)	11 (9.5–17)	7.6 (3.0–8.6)	<0.001
OSI	7.6 (6.5–11.5)	24 (22–24)	7.3 (5.6–15)	4.6 (1.9–5.0)	<0.001

AGO: Agomelatine; IRI: Ischemia reperfusion injury; TBARS: Thiobarbituric Acid Reactive Substances (nmol/g wet tissue); GSH: Reduced Glutathione (nmol/g wet tissue); CAT: Catalase (U/g protein); GSHPx: Glutathione Peroxidase (U/g protein); Total Nitrite: nmol/g wet tissue; TAS: Total Antioxidant Status (mmol Trolox Eqv/L); TOS: Total Oxidant Status (µmol H_2_O_2_ Eqv/L); OSI: Oxidative Stress Index (AU).

**Table 2:** Biochemical parameters measured in the serum of the different experimental groups.

Parameters	Sham	IRI	IRI-AGO	AGO-IRI	*p*
L-Arginine	0.9 (0.7–1.0)	0.7 (0.7–0.8)	0.8 (0.7–1.2)	1.6 (1.0–1.7)	0.030
ADMA	0.2 (0.2–0.2)	0.4 (0.3–0.4)	0.2 (0.2–0.2)	0.1 (0.1–0.2)	<0.001
SDMA	0.4 (0.4–0.6)	0.6 (0.5–0.7)	0.7 (0.6–0.9)	0.4 (0.2–0.5)	0.001
AST	242 (152–344)	470 (195–1344)	453 (344–1289)	244 (155–871)	0.260
ALT	79 (78–103)	237 (66–532)	221 (174–553)	144 (76–282)	0.190
ALP	166 (93–327)	419 (360–465)	210 (167–302)	342 (191–458)	0.010
GGT	4 (4–6)	4 (4–10)	4 (4–4)	4 (4–4)	0.500
LDH	1684 (1227–1728)	1923 (1504–4255)	2342 (1531–6029)	1424 (1243–3800)	0.370
TNF-α	216 (141–336)	310 (244–453)	243 (168–374)	307 (260–330)	0.390
IL-6	11.3 (6.0–14.4)	41.2 (30.2–45.4)	13.3 (9.6–27.5)	25.2 (13.0–45.1)	0.002
IL-1β	20.6 (18.3–44.2)	41.4 (27.7–50.9)	24.4 (18.8–31.4)	34.9 (28.5–48.7)	0.050

L-Arginine: µmol/L; ADMA: µmol/L; SDMA: µmol/L; AST: IU/L; ALT: IU/L; ALP: IU/L; GGT: IU/L; LDH: IU/L; TNF-α: ng/mL; IL-6: ng/L; IL-1β: pg/mL.

**Table 3:** Histopathological status of liver indicated by histopathological scores, the number of Kupffer cell and the density of caspase-3 positive cell in each group.

Parameters	Sham	IRI	IRI-AGO	AGO-IRI
Congestion	0 (0–0)	0 (0–3) ^a^	0 (0–1) ^b^	0 (0–2) ^e^
Necrotic hepatocytes	0 (0–1)	1 (0–3) ^a^	0 (0–3) ^b^	0 (0–2) ^e^
Glycogen loss	1 (0–1)	2 (0–3) ^a^	2 (0–3) ^c^	2 (0–3)
Kupffer cell	3 (1–8)	3 (1–8)	4 (1–9) ^d^	3 (1–10) ^f^
Caspase-positive cell	0 (0–1)	0 (0–3) ^a^	1 (0–2)	0 (0–2) ^f^

^a^*p* < 0.05 (IRI vs. Sham); ^b^ *p* < 0.05 (IRI+AGO vs. IRI); ^c^ *p* < 0.05 (IRI-AGO vs. AGO+IRI), ^d^ *p* < 0.05 (IRI-AGO vs. other groups), ^e^ *p* < 0.01 (AGO-IRI vs. IRI), ^f^ *p* < 0.01 (AGO-IRI vs. IRI +AGO). Different superscripts indicate statistically significant differences between the two groups (After the difference was determined in Kruskal-Wallis test. The Conover Test was performed to show which groups were different).

## Data Availability

The data supporting the findings of this study are available from the corresponding author upon reasonable request. The datasets were fully analyzed and reported within the manuscript, and no additional datasets beyond those presented are available in a public repository.
